# When Metabolism Recovers but the Brain Does Not: A Case Report of Posterior Reversible Encephalopathy Syndrome (PRES)-Like Encephalopathy After Extreme Metformin-Associated Lactic Acidosis

**DOI:** 10.7759/cureus.105129

**Published:** 2026-03-12

**Authors:** Anna-Katharina Eser, Rufat Mammadli, Maximilian Habs, Tobias Schmidt-Wilcke, Joachim Schessl

**Affiliations:** 1 Department of Neurology, Bezirksklinikum Mainkofen, MedizinCampus Niederbayern (MCN) University of Regensburg, Mainkofen, DEU

**Keywords:** cortical visual impairment, metformin-associated lactic acidosis, neurological manifestations, posterior reversible encephalopathy syndrome, toxic-metabolic encephalopathy

## Abstract

Metformin-associated lactic acidosis (MALA) is a rare but life-threatening complication of metformin therapy. Neurological manifestations are usually considered reversible and are classically associated with basal ganglia involvement on magnetic resonance imaging (MRI).

A 63-year-old man with previously normal renal function developed severe MALA after continuing metformin during a gastrointestinal illness. He presented with profound metabolic acidosis (pH 6.55), extreme lactate elevation to 29.9 mmol/L, acute kidney injury, and hemodynamic collapse requiring resuscitation. Toxic metformin levels confirmed the diagnosis. Despite rapid metabolic stabilization with renal replacement therapy, persistent encephalopathy remained. Serial MRI revealed bilateral cerebellar and posterior cortical vasogenic edema with contrast enhancement, without diffusion restriction, consistent with a posterior reversible encephalopathy syndrome (PRES)-like pattern. Severe cortical visual impairment persisted.

This is the first detailed report describing a PRES-like radiological pattern in the setting of confirmed severe MALA without the classical lentiform fork sign. Furthermore, we report a survivor of an initial lactate level of 29.9 mmol/L - one of the highest lactate concentrations ever reported in a surviving patient. This case challenges the assumption that neurological manifestations of MALA are uniformly reversible and expands the radiological spectrum of metformin-associated brain injury.

MALA may be associated with delayed and potentially irreversible toxic-metabolic brain injury despite rapid metabolic recovery. PRES-like imaging patterns can occur in metformin toxicity even in the absence of typical basal ganglia findings. This case report should raise awareness of this serious complication, encourage early neuroimaging in patients with persistent neurological symptoms, and emphasize prevention through appropriate sick-day management.

## Introduction

Metformin is the most widely prescribed first-line pharmacological treatment for type 2 diabetes mellitus and is generally considered safe when used appropriately [1,2]. A rare but potentially fatal adverse event is metformin-associated lactic acidosis (MALA), which typically occurs in the presence of renal impairment, hypoxia, sepsis, or hemodynamic instability. Although the metabolic derangement in MALA can often be rapidly corrected with renal replacement therapy, neurological complications are usually regarded as nonspecific and reversible [3-5].

Metformin-associated encephalopathy (MAE) has been described primarily in patients with renal failure and is most commonly associated with the characteristic *lentiform fork sign* on magnetic resonance imaging (MRI) [6]. However, structured reports of persistent or irreversible brain injury related to metformin toxicity remain scarce. Posterior reversible encephalopathy syndrome (PRES) is a clinicoradiological entity characterized by vasogenic cerebral edema, typically associated with hypertension, renal failure, sepsis, or toxic exposures [7]. PRES-like imaging patterns in the context of MALA have not been previously reported.

We present a case of extreme MALA in a patient without prior renal disease who developed delayed, PRES‑like neuroimaging abnormalities and persistent encephalopathy, even as metabolic parameters rapidly normalized.

## Case presentation

A 63-year-old man with a history of type 2 diabetes mellitus, arterial hypertension, and hypercholesterolemia was treated with metformin 1,000 mg twice daily, empagliflozin, sitagliptin, a fixed antihypertensive combination, and atorvastatin. Renal function had been normal in repeated outpatient follow-up examinations. Collateral history revealed inconsistent metformin intake, including autonomous dose adjustments and continued use during periods of gastrointestinal symptoms.

One day before admission, the patient developed nausea and diarrhea. On the day of admission, he experienced a sudden onset of severe dyspnea and tachypnea while at work. Initial evaluation by emergency medical services showed an oxygen saturation of 96%, which rapidly deteriorated to 80%, accompanied by reduced consciousness and bradycardia. Blood pressure was markedly elevated (210/100 mmHg).

Given the acute respiratory failure and suspected right heart involvement, fulminant pulmonary embolism (PE) was considered, and systemic thrombolysis with intravenous alteplase was administered prehospital by the emergency team. The patient was intubated and subsequently developed two episodes of pulseless electrical activity requiring cardiopulmonary resuscitation before sustained return of spontaneous circulation was achieved.

Arterial blood gas analysis on admission revealed extreme metabolic acidosis with a pH of 6.55 (normal 7.35-7.45) and a lactate level of 29.9 mmol/L (normal 0.5-2.0 mmol/L). Acute kidney injury (Kidney Disease Improving Global Outcomes (KDIGO) stage 3) was present, with a peak serum creatinine level of 5.65 mg/dL (normal range 0.6-1.3 mg/dL). Continuous renal replacement therapy was initiated and maintained for 48 hours.

**Figure 1:**
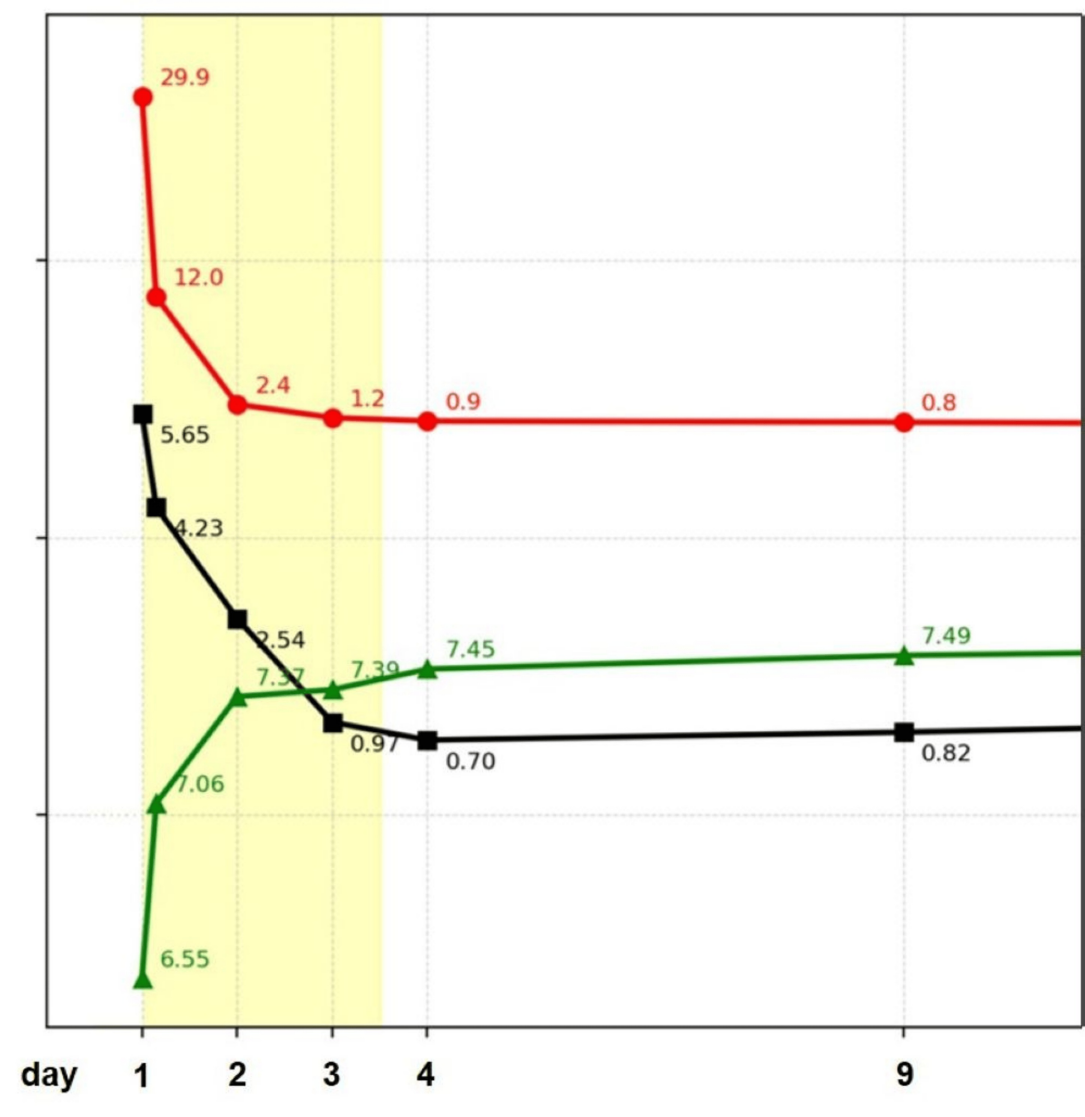
Temporal course of metabolic parameters. Temporal course of serum lactate in mmol/L (red), serum creatinine in mg/dL (black), and arterial pH (green) from day 1 to day 9. The yellow shaded area indicates the period of renal replacement therapy (dialysis). At presentation, markedly elevated lactate (29.9 mmol/L, normal 0.5-2.0 mmol/L) and creatinine (5.65 mg/dL, normal 0.6-1.3 mg/dL) levels were observed in the setting of severe metabolic acidosis (pH 6.55, normal 7.35-7.45). Following initiation of dialysis, there was a rapid decline in lactate and creatinine concentrations accompanied by normalization of arterial pH. During subsequent follow-up, parameters remained stable within the normal range.

Metabolic parameters improved rapidly under intensive care management, with normalization of lactate and pH values and subsequent recovery of renal function (Figure 1). In the context of severe lactic acidosis, acute kidney injury, and metformin exposure, MALA was diagnosed. This was confirmed by a toxic metformin serum concentration of 44.5 mg/L (therapeutic range: 1.0-5.0 mg/L). The patient was successfully extubated two weeks after admission.

Initial cranial computed tomography (CT) on admission showed no evidence of intracranial hemorrhage or acute ischemia. CT angiography excluded PE. Transthoracic echocardiography demonstrated transient right ventricular strain and mild pulmonary hypertension.

After sedation was reduced, delayed awakening was observed. Electroencephalography revealed generalized slowing without epileptiform activity, consistent with diffuse cerebral dysfunction. Somatosensory evoked potentials were preserved.

Follow-up cranial CT one week after admission demonstrated new bilateral cerebellar hypodensities. Brain MRI performed on day 12 showed bilateral cerebellar fluid attenuated inversion recovery (FLAIR) hyperintensities without diffusion restriction, indicating vasogenic edema. No hemorrhage or lentiform fork sign was detected.

Four weeks after admission, the patient was transferred to a neurological rehabilitation unit. He was awake but markedly slowed, disoriented, and cognitively impaired. Severe generalized weakness due to critical illness polyneuropathy/myopathy resulted in tetraparesis.

**Figure 2:**
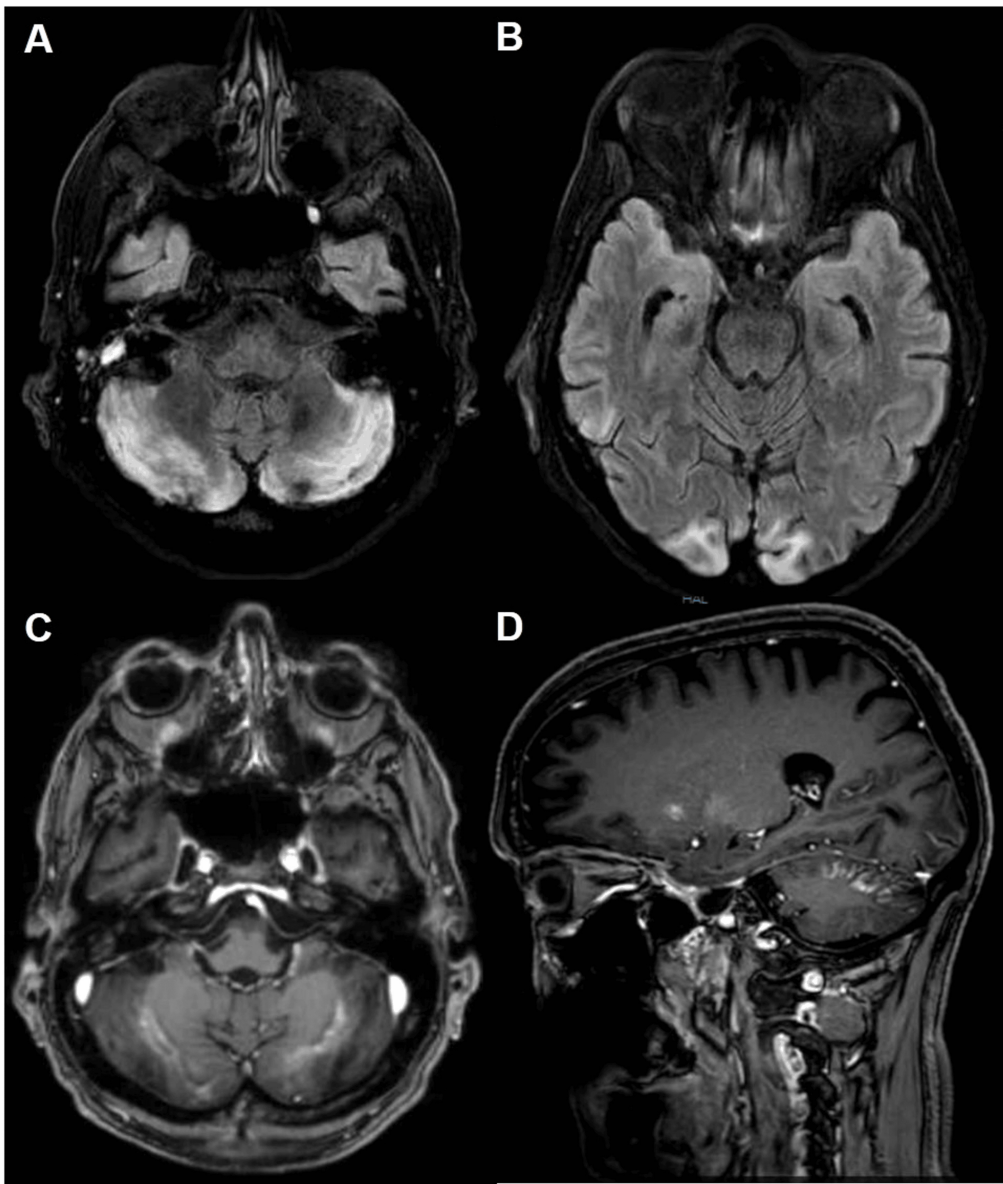
Follow-up brain MRI performed six weeks after admission. (A) T2w FLAIR axial images at the level of the medulla oblongata and the pons demonstrate persistent bilateral hyperintensities of the cerebellar hemispheres (B) with newly developed symmetric hyperintense lesions in the bilateral occipital poles. (C, D) Corresponding post-contrast T1-weighted images (axial and parasagittal) show pathological contrast enhancement within the cerebellar hemispheres and the occipital cortex, indicating ongoing blood-brain barrier disruption.

Multiple subsequent MRI examinations confirmed persistent cerebellar lesions with blood-brain barrier disruption and newly developed symmetric bilateral occipital and temporal FLAIR/T2 hyperintensities with contrast enhancements (Figure 2). Vascular imaging showed no evidence of vasculitis or relevant stenosis. The diagnosis of PRES was established based on the characteristic MRI findings, including bilateral, symmetric vasogenic edema predominantly affecting the parieto-occipital regions on FLAIR sequences without relevant diffusion restriction, in conjunction with the patient’s clinical history of renal failure.

Clinically, the patient developed severe central visual impairment. Visual evoked potentials were normal, and ophthalmologic examination revealed no retinal or optic nerve pathology, supporting a cortical origin of the visual deficit.

## Discussion

In this case, the diagnosis of MALA is unequivocal, supported by the highest lactate level of 29.9 mmol/L ever reported to our knowledge. Additionally, there was profound metabolic acidosis, acute renal failure, and hemodynamic collapse requiring resuscitation. In contrast, MAE is a poorly defined and rarely reported entity, described mainly in case reports and small case series [8-10]. Clinically, MAE typically presents with subacute disturbances of consciousness or cognition in temporal association with metformin exposure, often but not invariably accompanied by metabolic acidosis. Neuroimaging most commonly reveals symmetric vasogenic edema, which classically affects the basal ganglia (lentiform fork sign), although atypical localizations have been reported [6,11].

Conceptually, MALA and MAE should be distinguished. MALA represents a systemic metabolic disorder, whereas MAE reflects a toxic-metabolic brain injury, likely mediated by direct mitochondrial dysfunction with regional cerebral vulnerability [3,4,12-16]. The two conditions may occur simultaneously, but neither depends on the other. In the present patient, MALA is clearly established; whether MAE is also present remains uncertain. Although metabolic parameters normalized rapidly under renal replacement therapy, the patient developed persistent encephalopathy with progressive neuroimaging abnormalities.

The typical MRI finding is the lentiform fork sign, reflecting vasogenic edema of the basal ganglia [8,11,17,18]. In contrast, our patient exhibited cerebellar and posterior cortical involvement without basal ganglia lesions, consistent with a PRES-like pattern. Acute and reversible blindness is described in MALA [19,20]. A key feature of this case is the markedly delayed and prolonged evolution of neuroradiological findings. Initial MRI performed 12 days after the metabolic insult showed only subtle cerebellar FLAIR hyperintensities. In contrast, follow-up imaging weeks later demonstrated clear lesion progression with occipital involvement and contrast enhancement, still without diffusion restriction. This course differs from both classical hypoxic-ischemic brain injury and typical PRES, in which imaging abnormalities usually appear early and regress after control of triggering factors. The ultraprotracted and progressive pattern observed here suggests a sustained or secondarily evolving toxic-metabolic injury rather than an acute, self-limited process.

PRES represents an important differential diagnosis. PRES is commonly associated with acute hypertension, renal failure, sepsis, or immunosuppressive therapy and is characterized by symmetric parieto-occipital vasogenic edema without diffusion restriction [7]. In this case, acute renal dysfunction and an initial hypertensive crisis constituted potential PRES triggers.

However, the temporal profile is atypical for classical PRES. The progression of MRI abnormalities weeks after systemic stabilization, together with persistent clinical deficits, particularly visual impairment, argues against a primarily hypertension-driven PRES. Overall, a PRES-like imaging pattern in the setting of toxic-metabolic encephalopathy appears more plausible, although complete distinction is not possible due to overlapping clinical and radiological features.

Importantly, the patient had no known contraindications to metformin before this event. Continued metformin intake during a gastrointestinal illness with dehydration likely precipitated acute kidney injury and toxic accumulation. This highlights the critical importance of patient education regarding temporary discontinuation of metformin during intercurrent illness (*sick day rules*).

## Conclusions

MALA is a rare but life-threatening condition that may result not only in profound metabolic derangements but also in delayed and potentially irreversible neurological injury. Importantly, rapid correction of acidosis does not necessarily translate into neurological recovery. PRES-like imaging patterns may occur in metformin toxicity even in the absence of the classical lentiform fork sign, and their absence should not preclude consideration of metformin-related brain injury. Early brain MRI should therefore be considered in patients with persistent altered mental status following MALA. Greater awareness of this potential complication is essential, and strict adherence to sick day rules with prompt discontinuation of metformin during acute illness remains a key preventive strategy against this rare but devastating outcome.
